# Utility of Native T1 Mapping for the Evaluation of Myocardial Iron Overload in Patients with Thalassemia Major

**DOI:** 10.3390/tomography12040058

**Published:** 2026-04-14

**Authors:** Antonio Matteo Amadu, Alessio Contena, Alberto Dessì, Leandra Piscopo, Emma Solinas, Davide Turilli, Salvatore Claudio Fanni, Mariano Scaglione, Salvatore Masala

**Affiliations:** 1 Radiology Department of Surgery, Medicine and Pharmacy, University of Sassari, Viale S. Pietro, 07100 Sassari, Italyleandra.piscopo@gmail.com (L.P.); solinas.emma@gmail.com (E.S.);; 2 Department of Translational Research, Academic Radiology, University of Pisa, 56124 Pisa, Italy

**Keywords:** thalassemia major, myocardial iron overload, magnetic resonance imaging, T2*

## Abstract

**Purpose**: This study aimed to assess the utility of native T1 mapping for the evaluation of myocardial iron overload in patients with Thalassemia Major. T1 was compared to T2*, which represents the gold standard for iron quantification in the heart and liver. **Methods**: Consecutive patients with Thalassemia Major who performed cardiac MRI at the University Hospital of Sassari between 2022 and 2024 were prospectively included. All patients underwent a 1.5 T MRI with the same scanner (Philips Ingenia). T2* and native T1 mapping (MOLLI) sequences were performed in all patients on a mid-ventricular single 8 mm short-axis slice of the left ventricle. A region of interest was manually drawn in the septal wall. A T2* value < 20 ms was considered indicative of significant myocardial iron overload. A normal lower limit value of 990 ms was adopted for native T1 mapping. **Results**: In total, 100 patients with Thalassemia Major were included (median age, 45 [range, 7–80] years; 55% were male). The median myocardial T2* value was 31.4 (range, 5.1–47) and median T1 was 941 ms (range, 557–1131). A total of 12 patients (12%) exhibited T2* values < 20 ms; the T1 values in these patients (median, 733.8 ms [range, 557–975]) were significantly lower compared to those with a T2* of 20 ms or greater (median, 961 ms [range, 820–1131]), *p* < 0.001. No patient with T2* < 20 ms had a T1 value greater than or equal to 990 ms. Among the 88 patients with T2* ≥ 20 ms, 56 (64%) had T1 < 990 ms (median, 939.2 ms [range, 820–986]). Using a T1 threshold of 990 ms, the sensitivity was 100%, but the specificity was only 36%. ROC analysis identified an optimal T1 value of 895.5 ms, corresponding to 92% sensitivity and 100% specificity. **Conclusions**: Native T1 mapping is highly sensitive for detecting myocardial iron overload in Thalassemia Major, but the standard 990 ms threshold generates many false-positive results. In our cohort, adopting an ROC-optimized threshold of 895.5 ms markedly improved specificity while preserving excellent sensitivity.

## Simple Summary

Thalassemias are a heterogeneous and inherited genetic disorder characterized by the decreased synthesis of hemoglobin and anemia. The myocardial iron overload is an important consequence in people affected with thalassemia. Magnetic Resonance Imaging is currently the gold standard for measuring iron levels in the myocardium and liver. Magnetic Resonance Imaging is optimal for quantifying the iron load by the T2* relaxation time, which decreases in proportion to the rise of iron in the tissue.

## 1. Introduction

Thalassemias are a heterogeneous and inherited group of genetic disorders characterized by the decreased synthesis of hemoglobin (mainly alpha and beta chains) and by anemia that, in severe cases, is detected since childhood [1,2,3].

The most common way to classify thalassemia is based on the necessity of the blood transfusion support: group one, comprising patients who require regular transfusion or “Transfusion Dependent” (TD), and group two, comprising patients requiring occasional transfusions or “Non-Transfusion Dependent” (NTD) [3,4,5].

Beta-thalassemia is the most common hemoglobinopathy in Italy, with approximately 12 cases per 100,000 inhabitants (about 12,500 of patients have beta-thalassemia) and 3 million healthy carriers. Overall, roughly 6500 are TD, while 6000 are NTD. The Italian regions in which the disease is highly prevalent are Sicily, with 2700 cases, and Sardinia, with 1100 cases [3,4,5,6,7].

Other than anemia, myocardial iron overload (MIO) represents an important consequence in individuals affected by thalassemia major (TM). This condition arises from two main mechanisms. In TD patients, MIO is primarily associated with the iron contained in the blood given during regular transfusions, used to manage anemia [8,9]. In NTD individuals, instead, iron overload results mainly from the excessive intestinal absorption of iron [10,11,12].

Because of the risk of MIO and its clinical consequences, these patients undergo frequent Magnetic Resonance Imaging (MRI) examinations to measure iron levels in the myocardium and liver [13,14,15,16]. MRI is optimal for quantifying the iron load because in myocardial cells, it makes the transverse relaxation time faster, which could be detected by calculating the T2* relaxation time, which decreases in proportion to the rise in iron in the tissue. Consequently, a direct estimate of IO is obtained.

Cardiac T2* MRI is considered the gold standard for monitoring MIO because of its high sensitivity [17,18,19,20]. Diagnostic thresholds for T2* established in previous studies indicate that values greater than 20 ms are generally considered indicative of the absence of significant MIO. Values between 10 and 20 ms are associated with mild to moderate iron accumulation, whereas values lower than 10 ms indicate a very high risk of developing heart failure. In particular, when T2* values fall below 6 ms, nearly 50% of patients may develop heart failure within one year [17,18,19,20,21,22,23].

Due to its high sensitivity, the cardiac T2* MRI can predict cardiac dysfunction before it becomes clinically relevant or typical echocardiographic signs appear. Additionally, a cardiac T2* value of less than 20 ms has been related to a high risk of ventricular and supraventricular arrhythmias, particularly regarding atrial involvement and substrate characterization, including atrial fibrillation and atrial flutter [17,18,19,20,21,22,23].

In addition to this, another advantage of MRI is its multi-parametric features. In fact, the basic protocol also includes cine sequences, such as steady-state free precession (balanced SSFP), which analyzes ventricular volumes in different phases of the cardiac cycle, giving information about the main indices of cardiac function. In particular, the left ventricular ejection fraction (LVEF) is the percentage of blood ejected from the ventricle during each heartbeat. For this reason, it is an indicator of the overall cardiac function [24,25].

In patients with an important decrease in T2*, the consequences of MIO on ventricular function are marginal until the critical point is reached and quick worsening of cardiac function is observed. Therefore, regular monitoring with T2* MRI and the evaluation of LVEF are complementary approaches for early diagnosis and in monitoring complications, showing a complete view of myocardial status in patients with TM [26,27,28]. 

Despite this, iron overload cardiomyopathy, which causes 71% of deaths in TM patients, causes dilated cardiomyopathy, pericarditis, and heart dysfunction [29,30].

Moreover, recent advances in cardiac MRI have introduced native T1 mapping as a complementary technique to T2* for assessing myocardial tissue characteristics [31,32,33]. Unlike T2*, which primarily detects iron-related changes in transverse relaxation, T1 mapping measures longitudinal relaxation times and is sensitive to a broader range of myocardial alterations, including fibrosis, edema, and iron accumulation [32]. T1 mapping allows for the pixel-wise quantification of tissue properties, offering a more detailed evaluation of myocardial involvement and potentially improving the detection of changes in TM patients.

### Objective of the Study

Multiple studies demonstrated that T2* measurements may be affected by magnetic susceptibility artifacts and may be less sensitive in the early stages of iron accumulation [17,21,22,26]. In this context, native T1 mapping has emerged as a promising complementary technique for detecting myocardial tissue alterations [32,33,34,35,36], or in addition, could represent a valid alternative when T2* is not available [37,38].

Recently, progress on the T1 mapping method has widened the chances for the diagnosis of cardiac tissue disease. Furthermore, these methods are useful in the diagnosis and in defining prognosis in cardiac amyloidosis, myocarditis, and myocardial ischemia [32,33]. In addition to this, it is known that MIO causes the reduction in the relaxation time in T1 and T2*, showing a direct effect on both parameters [33,34,35,36].

Previous studies had already demonstrated that native T1 mapping and T2* are complementary [21,26,35,36].

The main aim of this study is to evaluate the added value of native T1 mapping in combination with T2 for detecting and monitoring MIO. Specifically, the study investigates whether T1 mapping can improve the early identification of myocardial changes, define specific diagnostic thresholds for T1 in thalassemia patients, and provide a reliable alternative or adjunct when T2 is limited due to artifacts or technical constraints. By establishing reference values and assessing the clinical impact of T1 mapping, this study seeks to enhance risk stratification and guide management strategies in patients with TM.

We evaluated the efficacy of native T1 mapping and T2* in detecting and monitoring CIO and its impact on clinical practice. Another aim of this study is to define the thresholds in T1 that could be useful in diagnosing CIO and compare them with those of T2* mapping [35,36,37].

## 2. Materials and Methods

Consecutive patients with TM who performed cardiac MRI at the University Hospital of Sassari between 2022 and 2024 were prospectively included. All participants underwent cardiac MRI scans using a system of 1.5 T (Philips Ingenia, Philips Medical Systems Veenpluis 6, 5684 PC Best, The Netherlands) with standard protocols. The acquisitions included multi-slice images directed in principal planes of the space, useful as a preliminary observation. Therefore, cine gradient-echo balanced steady-state free precession sequences (bSSFPs) were obtained and directed in classical visual in two, three, and four chambers, and short-axis images were acquired parallel to the mitral valve annulus, extending to cover the entire left ventricle from the base to the apex. All parametric maps were obtained during diastole in the same short-axis section at the mid-ventricular level. All MRI data were analyzed using a Philips workstation (*Cardiac Analysis Workflow*, *Philips Medical Systems*, *Ingenia*); T2* and T1 values were evaluated by manually drawing a region of interest (ROI) in the mid-septum. Care was taken to exclude epicardial structures, the insertion points of the right ventricle, and blood pool from the contours. A normal lower limit value of 990 ms for native T1 mapping was adopted in our center [32,33,36]. This value was derived from a cohort of 84 healthy subjects (median age 39 years [range: 12–70 years], 53% males). Evaluation was performed by two blind readers (with 6 and 4 years of cardiac MRI experience, respectively).

Analysis and cut-off optimization were performed with Jamovi (v 2.6). Descriptive parameters (mean ± SD; median, range) were calculated for age, native T1 mapping, and myocardial T2*. Normality was checked with the Shapiro–Wilk test. Diagnostic agreement between the conventional threshold T1 < 990 ms, and the reference standard T2* < 20 ms was evaluated using χ^2^ with continuity correction, Cramér’s V, and Cohen’s κ. A binary logistic regression model, with T2* < 20 ms as the dependent variable and continuous T1 as the predictor, provided model deviance, Nagelkerke R^2^, classification accuracy, and the area under the receiver operating characteristic curve (AUC). The ROC curve, built in Jamovi, was used to identify the optimal T1 cut-off by maximizing the Youden index. Statistical significance was set at *p* < 0.05.

## 3. Results

**Figure 1:**
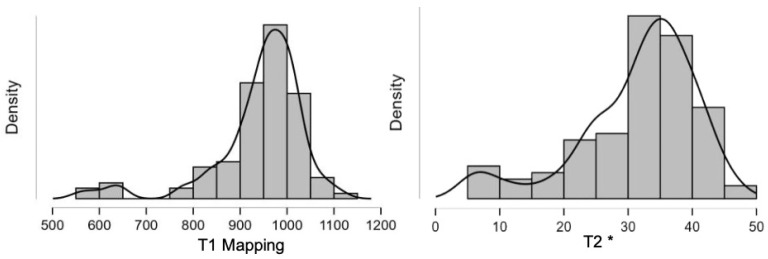
Distribution of native T1 mapping and T2* in our population.

**Figure 2:**
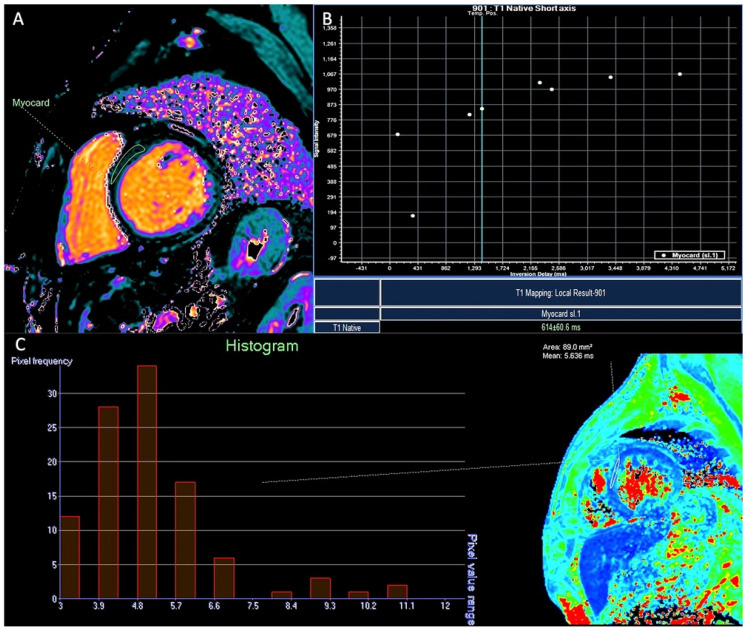
Example of patient who showed native T1 mapping < 990 ms (**A**,**B**) and T2* < 20 ms (**C**).

**Figure 3:**
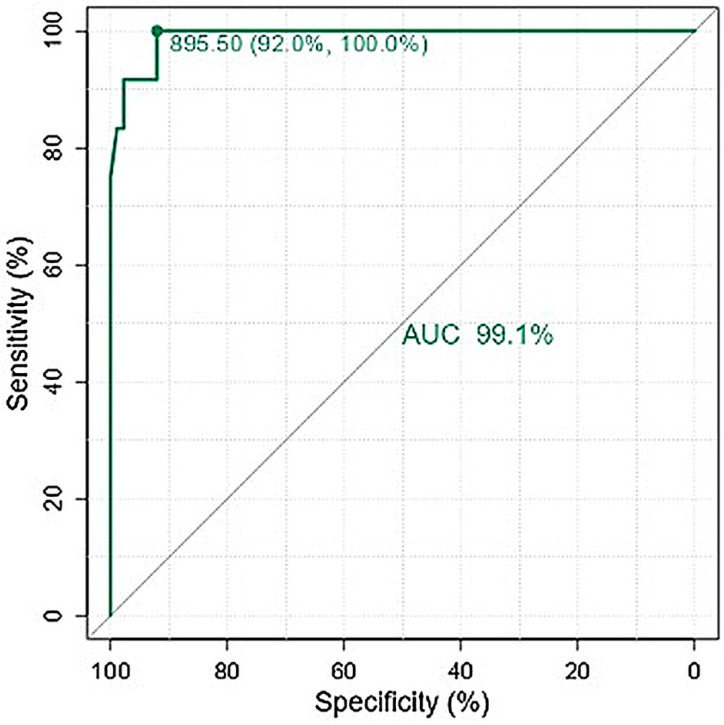
ROC analysis identified an optimal native T1 threshold of 895.5 ms.

One-hundred patients with TM were included (median age, 45 [range, 7–80] years; 55% were male). The median myocardial T2* value was 31.4 (range, 5.1–47) and the median T1 was 941 ms (range, 557–1131) (Figure 1). None of the continuous variables followed a Gaussian distribution (Shapiro–Wilk *p* < 0.001). Twelve patients (12%) exhibited T2* values < 20 ms; the T1 values in these patients (median, 733.8 ms [range, 557–975]) were significantly lower compared to those with a T2* of 20 ms or greater (median, 961 ms [range, 820–1131]), *p* < 0.001. No patient with T2* < 20 ms had a T1 value greater than or equal to 990 ms (Figure 2). Among the 88 patients with T2* ≥ 20 ms, 56 (64%) had a T1 < 990 ms (median, 939.2 ms [range, 820–986]). Using the T1 threshold of 990 ms, the sensitivity was 100% but the specificity was only 36%. Whether these “false positives” could represent subclinical iron deposition, fibrosis, or mixed tissue effects, rather than pure measurement error, in this context, further studies need to be conducted to support these results. The χ^2^ test confirmed a significant association between the two classifications (*p* = 0.011), yet the agreement was slight, with Cohen’s κ = 0.121. Logistic regression demonstrated a significant inverse relationship between T1 and the probability of MIO (*p* < 0.001); the model yielded a Nagelkerke R^2^ of 0.852, an AUC of 0.991, and an overall accuracy of 96% (sensitivity 83.3%, specificity 97.7%). ROC analysis identified an optimal T1 threshold of 895.5 ms, which achieved 92% sensitivity and 100% specificity, with an unchanged AUC of 0.991 (Figure 3). However, this threshold may not be directly transferable to other centers and external validation, and multicenter harmonization are required before clinical implementation.

## 4. Discussion

In patients with TM, cardiomyopathy secondary to iron overload remains the leading cause of mortality despite advances in chelation therapy [38,39,40,41]. MRI represents the only non-invasive method able to quantify myocardial iron deposition, with T2* relaxation time currently considered the reference standard [20,22,23]. However, T2* measurements may be affected by magnetic susceptibility artifacts and may be less sensitive in the early stages of iron accumulation [17,21,22,26]. In this context, native T1 mapping has emerged as a promising complementary technique for detecting early myocardial tissue alterations [32,35,36] or, in addition, could represent a valid alternative when T2* is not available [38].

Recent studies have increasingly highlighted the potential role of T1 mapping in the evaluation of MIO, suggesting that this technique may be sensitive to myocardial changes related to iron deposition and therefore provide complementary diagnostic information to conventional T2* [42,43,44,45,46].

Based on this assumption, the principal finding of the present study is the identification of a reduction in the native T1 mapping threshold in thalassemic patients, compared with normal values registered in our center, which is 990 ms. The optimal T1 threshold for these patients was identified to be a value of 895.5 ms; this cutoff demonstrated excellent diagnostic performance in our cohort, achieving 92% sensitivity and 100% specificity, and was substantially lower than the conventional reference value of 990 ms, which is commonly used as the lower limit of normal native T1 values in our institution.

When applying the traditional 990 ms threshold, a large number of apparent false-positive cases were observed. In particular, a considerable proportion of patients with T2* values > 20 ms, typically considered within the normal range for myocardial iron content, showed T1 values below this conventional cutoff. Consequently, while the 990 ms threshold demonstrated excellent sensitivity, it resulted in low specificity (36%).

The interpretation of these findings requires particular attention. The large proportion of patients (88 cases) with T2* values > 20 ms showed native T1 values below the proposed threshold. This observation suggests that T1 mapping may detect early myocardial alterations related to iron accumulation that are not yet identifiable using conventional T2* measurements.

One possible explanation is that the conventional T1 threshold may not be sufficiently specific for thalassemia populations. However, a more promising interpretation is that reduced T1 values in patients with apparently normal T2* may reflect early or subclinical myocardial iron deposition, preceding the degree of iron accumulation required to produce measurable T2* shortening. In this context, T1 mapping may be more sensitive for detecting early myocardial tissue changes, allowing the identification of patients with initial stages of MIO.

These findings therefore suggest that the use of T1 mapping in addition to T2* may improve the early detection of myocardial iron accumulation in thalassemia patients. Importantly, our results extend those of previous studies that have focused on defining T1 thresholds for iron detection [38,46]. Beyond identifying an optimized cutoff value, our study highlights the potential role of T1 mapping as an early marker of subclinical myocardial iron accumulation, enabling the identification of patients who might otherwise be considered normal based only on T2* values. This observation is in line with recent evidence suggesting that T1 mapping may detect myocardial iron-related tissue alterations before measurable changes in T2* occur, supporting its potential role as an imaging biomarker of myocardial involvement in TM [43,44,45,46].

Multiple studies support our result. For example, Singh et al. supported the fact that T1 and T2* correlate well, and the T1 value was low (<850 ms), with T2* being less than 20 ms [38]. Moreover, Sado et al. reported a T1 cutoff of approximately 836 ms to differentiate thalassemia patients from healthy controls [47]. Similarly, Feng et al. described mean myocardial T1 values to be around 653 ms in patients with significant MIO [48]. Although the thresholds reported in the literature vary, they are broadly consistent with the lower cutoff identified in our analysis. These differences may reflect variability in scanner vendors, magnetic field strength, sequence parameters, post-processing techniques, and heterogeneity in patient populations and iron burden.

From a clinical perspective, these findings suggest that native T1 mapping may provide important complementary information in the evaluation of MIO in thalassemia patients. While T2* imaging remains the reference standard for quantifying myocardial iron deposition, T1 mapping may improve the detection of subtle myocardial changes, particularly in patients with borderline or normal T2* values. In this scenario, T1 mapping could potentially be used as a screening or complementary tool, where a normal T1 value may help exclude clinically significant MIO, whereas reduced T1 values may identify patients who require closer monitoring or earlier adjustment of chelation therapy.

Another relevant implication of our findings concerns the interpretation of the traditional 990 ms threshold. In our cohort, this value showed high sensitivity but limited specificity, leading to a high number of apparent false-positive results. While these findings may partly reflect measurement variability or non-iron-related myocardial alterations, they may also indicate that the currently used threshold is too conservative for thalassemia populations. The lower cutoff identified in our study may therefore improve the specificity of T1 mapping when evaluating myocardial iron overload, while still preserving high sensitivity for detecting clinically relevant disease.

Different limitations of this study should be acknowledged. First, this was a single-center study; larger multicenter studies will be necessary to validate the proposed threshold and to assess its reproducibility across different populations and clinical settings. Second, the conventional normal lower limit for native T1 used in this study (990 ms) was derived from a local healthy control cohort, as previously reported in our study. Because native T1 values may vary between institutions, the use of locally derived reference ranges is common practice in cardiac MRI studies. Nevertheless, further characterization of the control population, including demographic information such as sample size and age distribution, would help to better contextualize the reference value and strengthen the interpretation of the results. Third, native T1 values are known to be influenced by several technical factors, including scanner vendor, magnetic field strength, and sequence design. For this reason, the optimized threshold of 895.5 ms identified in our study should not be considered universally applicable but rather specific to the scanner and imaging protocol used in our center. Differences in acquisition parameters across institutions may lead to systematic variations in absolute T1 values, highlighting the importance of center-specific calibration and multicenter harmonization before the adoption of standardized thresholds.

## 5. Conclusions

In conclusion, our results support the growing evidence that native T1 mapping provides incremental diagnostic information beyond conventional T2* imaging in the evaluation of MIO in thalassemia patients. The identification of a lower and more specific T1 threshold may improve the diagnostic accuracy of this technique and reduce false-positive findings when evaluating this population. At the same time, reduced T1 values in patients with normal T2* measurements may potentially reflect early myocardial alterations, highlighting the need for further longitudinal studies to clarify the role of T1 mapping in the early detection of myocardial iron accumulation.

## 6. Limitations

There were some limitations of our study. The group of patients with T2* < 20 ms was limited. No histopathological correlation was performed in our case for myocardial iron deposition. Another limitation was that there was no follow-up of patients. Future studies with long-term follow-up are required to establish the prognostic significance of native T1 mapping for myocardial iron deposition.

## Data Availability

The data presented in this study are available upon request from the corresponding author. The data is not publicly available due to privacy restrictions.

## References

[1] Taher A.T., Weatherall D.J., Cappellini M.D. (2018). Thalassaemia. Lancet.

[2] Weatherall D.J. (2018). The Evolving Spectrum of the Epidemiology of Thalassemia. Hematol. Oncol. Clin. N. Am..

[3] Viprakasit V., Ekwattanakit S. (2018). Clinical Classification, Screening and Diagnosis for Thalassemia. Hematol. Oncol. Clin. N. Am..

[4] Cappellini M.D., Cohen A., Porter J., Taher A., Viprakasit V. (2014). Guidelines for the Management of Transfusion Dependent Thalassaemia (TDT).

[5] Weatherall D.J. (2012). The definition and epidemiology of non-transfusion-dependent thalassemia. Blood Rev..

[6] Motta I., Bou-Fakhredin R., Taher A.T., Cappellini M.D. (2020). Beta Thalassemia: New Therapeutic Options Beyond Transfusion and Iron Chelation. Drugs.

[7] Cao A., Rosatelli C., Galanello R., Monni G., Olla G., Cossu P., Ristaldi M.S. (1989). The prevention of thalassemia in Sardinia. Clin. Genet..

[8] Saliba A., Taher A. (2015). Iron overload in transfusion-dependent thalassemia. Hematology.

[9] Ganz T., Nemeth E. (2023). Pathogenic Mechanisms in Thalassemia II: Iron Overload. Hematol. Oncol. Clin. N. Am..

[10] Taher A.T., Viprakasit V., Musallam K.M., Cappellini M.D. (2013). Treating iron overload in patients with non-transfusion-dependent thalassemia. Am. J. Hematol..

[11] Bou-Fakhredin R., Bazarbachi A.H., Chaya B., Sleiman J., Cappellini M.D., Taher A.T. (2017). Iron Overload and Chelation Therapy in Non-Transfusion Dependent Thalassemia. Int. J. Mol. Sci..

[12] Musallam K.M., Cappellini M.D., Wood J.C., Taher A.T. (2012). Iron overload in non-transfusion-dependent thalassemia: A clinical perspective. Blood Rev..

[13] Wood J.C. (2011). Impact of iron assessment by MRI. Hematol. Am. Soc. Hematol. Educ. Program.

[14] Fernandes J.L. (2018). MRI for Iron Overload in Thalassemia. Hematol. Oncol. Clin. N. Am..

[15] Ning X., Tan S., Peng F., Luo C., Tang C., Xiao F., Peng P. (2024). Organ-Specific Iron Overload in Non-Transfusion-Dependent Thalassemia Patients: Insights from Quantitative MRI Evaluation. Eur. J. Radiol..

[16] Hosseini-Bensenjan M., Haghpanah S., Sayadi M., Eghtedari M. (2023). Correlation of Serum Ferritin Level with Heart T2 MRI in Transfusion Dependent Thalassemia: A Systematic Review and Meta-Analysis. Clin. Lab..

[17] Jabbour J.P., Palombi M., Bonanni M., Matteucci A., Arcari L., Pierucci N., La Fazia V.M., Lavalle C., Mariani M.V. (2025). The Role of Cardiac Magnetic Resonance in Characterizing Atrial Cardiomyopathy and Guiding Substrate Ablation in Atrial Fibrillation: A Narrative Review. J. Cardiovasc. Dev. Dis..

[18] Anderson L.J., Holden S., Davis B., Prescott E., Charrier C.C., Bunce N.H., Firmin D.N., Wonke B., Porter J., Walker J.M. (2001). Cardiovascular T2-star (T2*) magnetic resonance for the early diagnosis of myocardial iron overload. Eur. Heart J..

[19] Brendel J.M., Kratzenstein A., Berger J., Hagen F., Nikolaou K., Gawaz M., Greulich S., Krumm P. (2023). T2* map at cardiac MRI reveals incidental hepatic and cardiac iron overload. Diagn. Interv. Imaging.

[20] Triadyaksa P., Oudkerk M., Sijens P.E. (2020). Cardiac T_2_* mapping: Techniques and clinical applications. J. Magn. Reson. Imaging.

[21] Messroghli D.R., Moon J.C., Ferreira V.M., Grosse-Wortmann L., He T., Kellman P., Mascherbauer J., Nezafat R., Salerno M., Schelbert E.B. (2017). Clinical recommendations for cardiovascular magnetic resonance mapping of T1, T2, T2* and extracellular volume: A consensus statement by the Society for Cardiovascular Magnetic Resonance (SCMR) endorsed by the European Association for Cardiovascular Imaging (EACVI). J. Cardiovasc. Magn. Reson..

[22] Chapchap E.C., Silva M.M.A., de Assis R.A., Kerbauy L.N., Diniz M.D.S., Rosemberg L.A., Loggetto S.R., Araujo A.D.S., Fabron Junior A., Verissimo M.P.A. (2023). Cardiac iron overload evaluation in thalassaemic patients using T2* magnetic resonance imaging following chelation therapy: A multicentre cross-sectional study. Hematol. Transfus. Cell Ther..

[23] Abedi I., Zamanian M., Bolhasani H., Jalilian M. (2023). CHMMOTv1—Cardiac and hepatic multi-echo (T2*) MRI images and clinical dataset for Iron overload on thalassemia patients. BMC Res. Notes.

[24] Solmaz H., Cabuk A.K., Altin Z., Albudak Ozcan E., Ozdogan O. (2021). Left ventricular systolic dyssynchrony index and endothelial dysfunction parameters as subclinical predictors of cardiovascular involvement in patients with beta-thalassemia major. Echocardiography.

[25] Klem I., Shah D.J., White R.D., Pennell D.J., van Rossum A.C., Regenfus M., Sechtem U., Schvartzman P.R., Hunold P., Croisille P. (2011). Prognostic value of routine cardiac magnetic resonance assessment of left ventricular ejection fraction and myocardial damage: An international, multicenter study. Circ. Cardiovasc. Imaging.

[26] Meloni A., Saba L., Cademartiri F., Positano V., Pistoia L., Cau R. (2024). Cardiovascular magnetic resonance in β-thalassemia major: Beyond T2. Radiol. Med..

[27] Chaosuwannakit N., Makarawate P., Wanitpongpun C. (2021). The Importance of Cardiac T2* Magnetic Resonance Imaging for Monitoring Cardiac Siderosis in Thalassemia Major Patients. Tomography.

[28] Kirk P., Roughton M., Porter J.B., Walker J.M., Tanner M.A., Patel J., Wu D., Taylor J., Westwood M.A., Anderson L.J. (2009). Cardiac T2* magnetic resonance for prediction of cardiac complications in thalassemia major. Circulation.

[29] Murphy C.J., Oudit G.Y. (2010). Iron-overload cardiomyopathy: Pathophysiology, diagnosis, and treatment. J. Card. Fail..

[30] Ansharullah B.A., Sutanto H., Romadhon P.Z. (2025). Thalassemia and iron overload cardiomyopathy: Pathophysiological insights, clinical implications, and management strategies. Curr. Probl. Cardiol..

[31] Puntmann V.O., Peker E., Chandrashekhar Y., Nagel E. (2016). T1 Mapping in Characterizing Myocardial Disease: A Comprehensive Review. Circ. Res..

[32] Kumfu S., Chattipakorn S.C., Chattipakorn N. (2022). Iron overload cardiomyopathy: Using the latest evidence to inform future applications. Exp. Biol. Med..

[33] Schelbert E.B., Messroghli D.R. (2016). State of the Art: Clinical Applications of Cardiac T1 Mapping. Radiology.

[34] Mavrogeni S.I., Gotsis E.D., Markussis V., Tsekos N., Politis C., Vretou E., Kermastinos D. (1998). T2 relaxation time study of iron overload in b-thalassemia. Magn. Reson. Mater. Phys. Biol. Med..

[35] Torlasco C., Cassinerio E., Roghi A., Faini A., Capecchi M., Abdel-Gadir A., Giannattasio C., Parati G., Moon J.C., Cappellini M.D. (2018). Role of T1 mapping as a complementary tool to T2* for non-invasive cardiac iron overload assessment. PLoS ONE.

[36] Krittayaphong R., Zhang S., Saiviroonporn P., Viprakasit V., Tanapibunpon P., Komoltri C., Wangworatrakul W. (2017). Detection of cardiac iron overload with native magnetic resonance T1 and T2 mapping in patients with thalassemia. Int. J. Cardiol..

[37] Meloni A., Martini N., Positano V., De Luca A., Pistoia L., Sbragi S., Spasiano A., Casini T., Bitti P.P., Allò M. (2021). Myocardial iron overload by cardiovascular magnetic resonance native segmental T1 mapping: A sensitive approach that correlates with cardiac complications. J. Cardiovasc. Magn. Reson..

[38] Singh S.P., Jagia P., Ojha V., Seth T., Naik N., Ganga K.P., Kumar S. (2023). Diagnostic Value of T1 Mapping in Detecting Iron Overload in Indian Patients with Thalassemia Major: A Comparison with T2* Mapping. Indian J. Radiol. Imaging.

[39] Aessopos A., Kati M., Tsironi M. (2008). Congestive heart failure and treatment in thalassemia major. Hemoglobin.

[40] Borgna-Pignatti C., Cappellini M.D., De Stefano P., Del Vecchio G.C., Forni G.L., Gamberini M.R., Ghilardi R., Origa R., Piga A., Romeo M.A. (2005). Survival and complications in thalassemia. Ann. N. Y. Acad. Sci..

[41] Bruzzese A., Martino E.A., Mendicino F., Lucia E., Olivito V., Bova C., Filippelli G., Capodanno I., Neri A., Morabito F. (2023). Iron chelation therapy. Eur. J. Haematol..

[42] Wang L.E., Muttar S., Badawy S.M. (2025). The challenges of iron chelation therapy in thalassemia: How do we overcome them?. Expert Rev. Hematol..

[43] Radenkovic D., Weingärtner S., Ricketts L., Moon J.C., Captur G. (2017). T_1_ mapping in cardiac MRI. Heart Fail. Rev..

[44] Reiter G., Reiter C., Kräuter C., Fuchsjäger M., Reiter U. (2018). Cardiac magnetic resonance T1 mapping. Part 1: Aspects of acquisition and evaluation. Eur. J. Radiol..

[45] Reiter U., Reiter C., Kräuter C., Fuchsjäger M., Reiter G. (2018). Cardiac magnetic resonance T1 mapping. Part 2: Diagnostic potential and applications. Eur. J. Radiol..

[46] Selim O.M.H.Z., Ibrahim A.S.A.H., Aly N.H., Hegazy S.N.A., Ebeid F.S.E. (2024). Early detection of myocardial iron overload in patients with β-thalassemia major using cardiac magnetic resonance T1 mapping. Magn. Reson. Imaging.

[47] Sado D.M., Maestrini V., Piechnik S.K., Banypersad S.M., White S.K., Flett A.S., Robson M.D., Neubauer S., Ariti C., Arai A. (2015). Noncontrast myocardial T1 mapping using cardiovascular magnetic resonance for iron overload. J. Magn. Reson. Imaging.

[48] Feng Y., He T., Carpenter J.P., Jabbour A., Alam M.H., Gatehouse P.D., Greiser A., Messroghli D., Firmin D.N., Pennell D.J. (2013). In vivo comparison of myocardial T1 with T2 and T2* in thalassaemia major. J. Magn. Reson. Imaging.

